# Cancer survivorship: existential suffering

**DOI:** 10.1080/17482631.2021.2001897

**Published:** 2021-11-13

**Authors:** Venke Ueland, Elin Dysvik, Jessica Hemberg, Bodil Furnes

**Affiliations:** a Faculty of Health Sciences, University of Stavanger, University of Stavanger, Stavanger, Norway; b Emerita Faculty of Health Sciences, University of Stavanger, Stavanger, Norway; c Faculty of Education and Welfare Studies, Åbo Akademi University, Vaasa, Finland; d Faculty of Health Sciences, University of Stavanger, Stavanger, Norway

**Keywords:** Cancer survivor, existential experience, phenomenological hermeneutics, intermediate state, enduring, transitions, lived experience, lifeworld, suffering

## Abstract

**Purpose:**

This study aimed to explore and describe existential experiences after cancer treatment.

**Method:**

An exploratory phenomenological hermeneutical design was used following in-depth interviews with 21 people.

**Results:**

The study revealed experiences of multifaceted suffering in the form of limitations in everyday life, inner struggles, and bearing the burden alone.

**Conclusions:**

Existential suffering after cancer treatment was revealed as like being in a process of transition, in an intermediate state, as moving between suffering and enduring, and alternating between alienworld and homeworld. A new and broader professional perspective is needed to establish rehabilitation services based on multifaceted experiences of suffering. This means a shift in focus from biomedical symptoms towards understanding of existential meaning for the person.

## Introduction

Finished cancer treatment? What then? More and more people are undergoing cancer treatment and cancer survival rates have increased substantially over the past decade. In Europe, approximately half of all patients diagnosed with cancer will survive for 10 years or more (Lagergren et al., 2019). When the primary treatment is completed, patients enter a transition period where they have to adapt to the rest of their lives (Wood, 2018; see also Hvidt, 2017; Fitch et al., 2019).

People want to return to the life they had before being affected by cancer, but many people find this experience difficult because their life power is limited (Hvidt, 2017; see also Ellingson, 2017). Earlier studies have highlighted the need to fill the gap in the current health-care approach to cancer survivors by focusing on survivors’ existential burden and ways to support the transition process towards becoming more whole as a person (Ueland, Dysvik et al., 2020; Ueland, Rørtveit et al., 2020).

## Background

Many people who have undergone cancer treatment are living with both the side effects and the long-term effects of their therapy (Ellingson, 2017; Ellingson & Borofka, 2020). Previous research has emphasized that different types of damage affect a range of everyday life activities such as social life, working life, finances, and relationships. Particularly pronounced are the symptoms of fatigue, exhaustion, and lack of concentration (Ellingson, 2017; Ellingson & Borofka, 2020; see also Fitch et al., 2019; Ueland, Dysvik et al., 2020). As such, the areas of life affected impose various limitations on everyday life (Hvidt, 2017; see also Knox, 2020a). Other studies reveal experiences of feeling significantly different after finishing cancer treatment (Ellingson & Borofka, 2020; Knox, 2018; Tindle et al., 2019). The whole person seems to be affected. It seems as though something inside the person is injured and needs healing (Knox, 2013, 2018) and that there is need for a framework of care to facilitate the transition from the experience of suffering to the experience of wholeness (MacDonald et al., 2021).

Existential challenges point to an experience of several losses, such as “not feeling like oneself” anymore, with an impact on living the ordinary life as before (Ueland, Dysvik et al., 2020; Ueland, Rørtveit et al., 2020; see also Hvidt, 2017; Tindle et al., 2019; Ellingson & Borofka, 2020; Knox, 2020a). Further existential challenges in patients with cancer could be described as escaping or remaining in the cage of inauthentic self (Khoshnood et al., 2018). Following cancer, patients are exposed to a greater awareness about themselves. They have changed their thoughts concerning oneself to those concerning inauthentic self, which triggers existential questions regarding the past and the fear of the future and experience a collapse of physical body identity (Khoshnood et al., 2018).

Studies indicate that when cancer treatment ends, patients enter a period where they must adapt to their new life on their own without any support from health-care providers. This in itself is demanding and for many, an exhausting situation (Hvidt, 2017). In addition, suffering patients sometimes feel they need to protect others from their suffering and therefore suppress it consciously, which has been called “doubled suffering” (Langegard & Ahlberg, 2009). The period after cancer treatment can intensify alienation and we believe that support from professionals is central to creating a more balanced life (Mikkelsen et al., 2008).

Hvidt (2017) raises the question of whether illness and suffering, as an existential phenomenon in the Western world has been “expropriated” from our health-care system. She calls for more research that explores how health-care providers might improve their communication skills regarding existential dimensions of illness.

Suffering is described as an unavoidable natural part of life and can be about everything from a threat to one’s total existence to a loss of opportunity to complete various social tasks. Life suffering is related to what it means to live, to be human among other people and refers to the human existence as a whole where experiencing suffering can mean not to be able to hold oneself together as a whole (Eriksson, 2018; Lindström et al., 2011). Moreover, the experiences of loss in one’s life situation is accompanied by an inner suffering which could be difficult to deal with. As such, these findings create a need for support and guidance (Furnes et al., 2015). Suffering is a unique experience that can only be known to the sufferer. Morse (2001, 2011) defines suffering as a basic human response to a physical or psychosocial threat. Two broad behavioural states in suffering, enduring and emotional suppression, are identified (Morse, 2011).

Research over the last decade has, to a large extent focused on side effects such as physical and psychosocial damage, emotions, coping, quality of life and how the workplace is experienced. Little research has been conducted on the impact surviving cancer has on human beings (Ueland et al., 2018; see also Ellingson & Borofka, 2020; Knox, 2020b;). Furthermore, little has been done towards understanding survivors’ fundamental sense of being in life “as having a palpable sense of temporality and mortality awakened in oneself” (Knox, 2020b). Lives of those affected by cancer are about so much more than having survived cancer, which suggests there is a need for more attention to be paid to the challenges that people may face when they have finished cancer treatment (Hvidt, 2013; see also Ueland, Dysvik et al., 2020).

Because research beyond late effect is sparse, we sought to gain increased knowledge of existential experiences using qualitative research interviews. As such, the aim of this study was to explore and describe existential experiences after cancer treatment.

## Methods

Our study had an exploratory phenomenological hermeneutical approach and involved qualitative interviews (Gadamer, 2007; Galvin & Todres, 2013). A lifeworld approach was used to gain a deeper understanding of the phenomenon under investigation (Dahlberg et al., 2008). Moreover, a first-person perspective was used and found to be helpful in considering the person’s experiential “views from inside” (Galvin & Todres, 2013). This approach was supported by Gadamer (2007) who emphasized that meaning is contextual because people understand themselves through the world in which they live.

Based on Kvale and Brinkmann (2009), an open lifeworld approach was outlined by unstructured, qualitative research interviews. We sought to introduce a lifeworld perspective focusing on how persons who had been treated for cancer experience their lives. We wanted to move beyond labels (e.g., depression, fatigue) to gain a better understanding of the existential experiences expressed by the interviewees.

### Data collection

We recruited participants among adults who had completed cancer treatment from a local Cancer Association, an organization that provides support, counselling and guidance to people affected by cancer. The data collection was based on a purposive sampling, as we chose patients who were well informed of the phenomenon of interest. Twenty-one people (6 men and 15 women) between 28 and 72 years of age, agreed to participate. The participants had all been affected by different types of cancer and treatments, including breast cancer, prostate cancer, colorectal cancer, gynaecological cancer, testicular cancer, Hodgkin’s lymphoma, and non-Hodgkin/CNS lymphoma. Most had finished cancer treatment at least 4 years before the study (Table I).

**Table I.** Background information of the participants who have received cancer treatment. There is no correspondence between the table listing and the number in the findings

Sex	Age (Years)	Occupation	Year of Treatment Completion
Female	35	Working	2016
Female	38	Sick leave	2019
Female	40	Working	2015
Female	42	Working	2018
Female	48	Working	2015
Female	50	Disability benefit	2017
Female	50	Working	2018
Female	52	Working	2015
Female	56	Sick leave	2016
Female	57	Working	2017
Female	57	Working	2009
Female	60	Working	2017
Female	65	Disability benefit	2016
Female	72	Retired	2015
Female	72	Retired	2017
Male	28	Working	2016
Male	42	Working	2010
Male	53	Working	2012
Male	53	Working	2016
Male	58	Disability benefit	2018
Male	64	Retired	2018

(N = 21)

The inclusion criteria were persons that had completed cancer treatment, were feeling healthy at the time of the study, were motivated to participate in an in-depth interview, and were willing to share their experiences. One patient had recently been informed about cancer metastases but was still interviewed because of his experiences of life after cancer treatment.

Participants were invited to join the study through the Cancer Association leader who provided written information to those who were in contact with the Cancer Association. Those who were interested in participating contacted the first author directly to ensure anonymity. After they had agreed to join the study, each participant contacted the first author to plan the location and time for the interview.

The interviews took place in a meeting room at the Cancer Association’s offices and were led by the first author. The interviews lasted for 60 to 90 minutes each. We strived for an open approach in the in-depth interviews. Nuances were explored by asking follow-up questions. Each interview started with the line, “Please tell me about your experiences after being treated for cancer.” The follow-up questions focused on the participant’s thoughts and feelings related to existential experiences in the context of their post-cancer situation. Openness towards the subject and phenomenon described, and an attempt to disregard prior knowledge were central to the approach. The interviews were audiotaped and transcribed verbatim. The transcriptions provided the empirical data material for this study.

### Data analysis

The text was analysed and interpreted on three levels: a self-understanding level; a common-sense level; and a theoretical level (Table II) (Kvale & Brinkmann, 2009). This process occurred as a hermeneutical movement between the particular and the whole, wherein the interpreter cannot be fully aware of his/her own prejudices and pre-understanding (Gadamer, 2007).

**Table II.** Examples of three contexts of interpretations

Three contexts of interpretations			
Self-understanding	Thematized findings	Common sense	Theoretical interpretation
*My energy level is completely changed. My shape varies a lot, bouncing up and down within one single day. There are so many limitations to the framework for my life. My body just doesn’t cope with some of the things I need to feel good about myself.*	*Limitation in everyday life*	*They experience living in a changed **living space**, where one lives with limitations*	The Intermediate state of transition: Suffering -enduring Alien world- Home world(the existential)
*So many things feel different now. I’m still in the process of getting to know my new self, finding the new me, because all areas of my life are affected. I’m constantly seeking to identify what I can do, to feel mastering and the joy of pushing myself a bit, but not pushing too far. If I let go now, will things then fall completely apart?*	*Inner struggle*	*They experience living in an **intermediate state**, which creates a varying degree of courage for life*. They wage a battle with themselves, struggling to find the courage to live	The Intermediate state of transition: Suffering -enduring Alien world- Home world
*They look upon me as if I’m the same, but I don’t feel entirely the same. I wish the attention they gave me had lasted longer, because I still feel ill. Others don’t understand, so I adapt. I let go of myself. It’s a strange life, a different life. I’m here and they’re outside. It feels as if I’m in another world.*	*Bearing the burden alone.*	*In the face of a lack of **resonance**, they experience a lack of self-understanding, which increases alienation*. They struggle to understand themselves, missing a room where they are met and understood by their surroundings	The Intermediate state of transition: Suffering -enduring Alien world- Home world

All authors were involved in the interpretation and all levels of analysis. The researchers’ pre-understanding involved the belief that existential phenomena are essential when struggling with health-related issues.

#### Self-understanding

The entire text was read and re-read, and an initial holistic understanding was derived, from which fragments of significance emerged. The interpretation aimed to reveal the participants’ self-understanding. Thereafter, meaning units were organized and arranged under preliminary headings. The co-authors participated in this process with discussion to reach consensus.

#### Common sense

The participants’ self-understanding was reformulated, a new level of abstraction emerged, and the units in the text were systematized and visualized as interconnected topics. This form of abstraction revealed three themes that are presented in the Results section.

#### Theoretical interpretation

Here, the analysis and interpretation are based on previous research and relevant theories that may deepen our understanding of the findings. This process is further presented in the Discussion section.

### Ethical considerations

The study was approved by the Ethical Committee and by the Norwegian Social Science Data Services (no 2018/2278). All participants provided their consent after receiving written information about the project. Confidentiality was guaranteed and participants were expressly advised of their right to withdraw from the study without prejudice and without needing to give any reason. Deidentified data were stored in accordance with strict protocols at the University of Stavanger and within the Ethical Committee. The participants were informed that they had access to professional support at the clinic after the interviews, if needed.

## Findings

The study’s thematized findings represent an interpretation of the participants’ expressions of their existential experiences following cancer treatment. The thematized findings are: *Limitations in everyday life, Inner struggle, Bearing the burden alone.*

We present the three thematized findings by putting together meaningful quotations from different participants and gathering these into one coherent text. This meaning condensate, based on composition of meaningful quotations, provides a rich and nuanced description of the topics highlighted in the empirical material (Kvale & Brinkmann, 2009).

In the following sections we present each of the three meaning condensates and after each presentation, we offer an interpretation on a common-sense level.

### Limitations in everyday life

All interviewees described significant limitations in their life following completion of cancer treatment. The limitations appear in several areas. They described their experiences in terms of becoming rapidly weary, lacking a zest for life and energy. They did not have the energy to travel, to attend parties, and to be as socially outward as they used to be. Their frame of life had become restricted, with the responsibility they have for themselves and their own happiness becoming a burden. They tended to compare their current situation with how they were able to live in the past, recognizing that they are unable to commit to life, that they must hold back their involvement.

My energy level has become completely changed. My current shape varies a lot, bouncing up and down within one single day. There are so many limitations to the framework for my life. I would long to be normal and that there were no limitations to my life, allowing me to feel passionate about something. Because when I’m passionate about something, it wears down my body; my body just doesn’t tolerate it. After the cancer treatment I have to live a much quieter life. Preferably, I also have to think and talk more slowly, which is opposite to my nature. That’s not who I am. I dare not live strongly anymore. Going to a cafe is good for my soul but being with lots of people makes me very tired. My body just doesn’t cope with some of the things I need to feel good about myself. I belong to the kind of people who have an urge to talk. I find all this has been difficult. Not having the energy. You don’t return to the one you were before, I’ve accepted that. Too many things have happened. I like living an active life, I like going to work, I like working out and things like that. But after my treatment was finished, I found I could never achieve flow. I’m living in my own little bubble. I’m not working properly. It’s not a coma, but a vacuum. I’m here, but I’m not working as a person. I’m unable to go to work. The way I see it, nothing about me is working properly. It’s like the old car batteries. You had to make sure not to drain the battery completely, because if you did that, it would be impossible to recharge it. That’s how I feel. I put myself on charging, but no charging occurs. I have no vitality, no energy. Less elastic, I think that was a very good description. I have trouble making future plans as it is now. The fear of getting cancer is much more present now that I’ve had cancer and seen that my body can produce it. There’s a small shadow beside me. I live much more in the present. I don’t want to be thinking about cancer all the time. I want to put an end to that, and not look back. From now on there’s only that way (pointing forward).

### Common-sense level

Participants’ living space was experienced as transformed, incomprehensible, limited, and burdensome. The diagnosis is a challenge in itself. Their body has let them down, being able to produce cancer cells. The diagnosis casts a weight on the life they have to live from now on. The participants are restricted by late effects, as well as the exhausting limitations that come from being different from the way they were. The limitations are an obstruction to their self-realization. As a result of his/her limitations, the person is unable to live the way he/she needs to feel well. The way they feel about themselves is not really who they are. It’s as if they have lost themselves after cancer treatment.

### Inner struggle

All participants gave their thoughts on finding the way ahead with their limitations: how to delimit their life, their relationships, choosing to socialize less, working reduced hours, or subsisting on disability benefits. Several of them say they have become more “selfish”, it is not something they wish to be, but they have more than enough to do with looking after themselves. Some say they have found it difficult to return to work, and some have been compelled to work part time to adapt to the demands of working life. Unable to cope with the things they managed before, an adjustment is required for which they are ultimately responsible. They needed to change in order to fit in and it is a responsibility they have to carry alone. It is a process of trying and failing, of struggling forward, of stumbling and rising again. The problem is, there is so much to make out, because they do not immediately realize how their state is, what the limitations are, and what they are able to take on. Many of them say they are struggling to find a way back to their place in a meaningful life. Carrying one’s health, carrying oneself, carrying the life they have now, feels like a responsibility.

I’ve accepted that I have to carry responsibility for my own health. So many things feel different now. I’m still in the process of getting to know my new self, finding the new me, because all areas of my life are affected. There’s so much loss and grief, so that I’m constantly seeking to identify what I can do, to feel mastering and the joy of pushing myself a bit, but not pushing too far. There’s a stigma attached to not being a resource, to being ill. It has been important for me to ensure that when I’m at work, I’m at work. If it doesn’t work out, I need to do something about it myself. In the event that it doesn’t, I’ll have to start working reduced hours. As long as I have a job, I need to perform. You need to keep certain things to yourself – to maintain your value. That’s why, I think, I’ve omitted to communicate to my surroundings the things that have been difficult. But it’s there, lurking; is it OK if I work reduced hours, is it because I’m lazy? I long for not having to justify myself, I just want to feel good. I’ve toiled a lot with my relations. Who means something to me? What is important in life? What will I use my energy and time on? It was always my goal to make it. I’ve fought my way up. If I let go now, will things then fall completely apart? I live much more in the present now, I appreciate the ordinary days more, I don’t need things to happen all the time. I’m just so happy being able to get up in the morning, not having to plan ahead or taking part in things I enjoyed before. Actually, I have trouble planning ahead. It’s uninteresting. It’s the here and now that count. I’ve found new ways; I don’t set any big goals. I’ve gradually managed to let go more and more.

### Common-sense level

Life is lived in an intermediate state, waging a battle against oneself, living within a constricted living space, a no-man’s land. They live on the side of life, struggling to make their new life work and find their way forward. The participants carry on living in their own way, living on the sideline, restricted in their self-realization. However, they do adapt and carry themselves and life further. They reflect on the possibilities of progressing personally now that they are changed, a dialogue on life and the road forward. It is a battle to move on, a struggle to find the courage to go on living. The participants are waging an inner battle with themselves to not lose themselves. There are traces of carrying oneself further in life, of moving on with life. A force is put into action. They strive to endure and cope with their restricted life, more than they try to expand it. It seems it is impossible to push their final limits. A life of varying courage is glimpsed. They feel a responsibility to carry themselves and their life, trying out and testing a way of living that may work for them. They set a new cautious compass course. Life consists in just existing and to endure the situation.

### Bearing the burden alone

All interviewees experienced a lack of understanding from their surroundings. They are considered as healthy and their illness is seen as a thing of the past, best forgotten. Everyone can be tired sometimes; life should go on. It is hard to explain oneself, and hard explaining to others how they are. They experience that their continuing struggle is unrecognized.

No, I don’t think they understand how it is. Everybody felt compassion for me during the year of treatment, but in the three years after there was no understanding. Everyone faces challenges in life, so it’s not like you’re a special case anymore. My friends run around as if nothing has happened. They look upon me as if I’m the same, but I don’t feel entirely the same. I’m not as vivacious as I was. I feel my illness has been trivialized. I wish the attention they gave me had lasted longer than the radiation period, because I still feel ill. «It’s so good that it’s over ». But it’s not over; it’s only for them that it’s over. It’s like having an invisible illness. Some friends I cannot even talk to about my illness. They’re simply not interested in hearing how I am. Maybe they’re thinking that I’m holding on to something? Others don’t understand, so I adapt. I let go of myself. It’s a strange life, a different life. I’m here and they’re outside. It feels as if I’m in another world.

### Common-sense level

The lack of resonance from the surroundings is a challenge, and so is living in the predominant culture and community with their experienced life situation. They do not find a room where they are received. The participants feel alienated and live life in a different sphere than the people around them. In a way it is like being shut off from the world; with them and their situation having no place in the community. It is a burdensome way to be in the world, they are a stranger to themselves, and they are not accepted. In the wake of cancer treatment, they are faced with a different person than the one they were. Having to go into the world feeling themselves to be different increases their alienation. It is a double suffering. They are a different person from the one they were, whereas in encounters with others, they are seen as they were, which enhances the distance they feel between themselves and others.

## Discussion

The aim of this study was to explore and describe existential experiences after cancer treatment.

The discussion will lead by interpretation and deepen our understanding of suffering, with reference to relevant theory and previous research. The thematized findings, *Limitation in everyday life, Inner struggle*, and *Bearing the burden alone*, guide the selection of theory.

In our study, lived experiences of cancer survivors reveal multifaceted and complex suffering. Suffering is obviously related to the limitations of everyday life. This means not being able to do what was significant before due to lack of life power, holding oneself back, trying not to be involved or engaged. As such, suffering may cause loss of life content. Loss of possibilities to unfold life as before is basically caused by curtailed vitality. This points towards an existential suffering, missing one’s prior self, constrained by being in the world in a limited way. In line with the French philosopher Merleau-Ponty (2002), a person’s embodiment may become broken when he/she experiences life-changing events, such as cancer. Our findings suggest that being a cancer survivor represents such brokenness because immediate being in the world can be lost (Merleau-Ponty, 2002; see also Fuchs, 2002;).

The American philosopher Steinbock (1995) describes how people can feel an existential homelessness after significant “breaks” in life. In our study, there seems to be a brokenness experienced by the participants because they feel unable to live fully in the familiar homeworld, as alienworld is foreign and different from their homeworld. They seem to be striving to interweave and unite these two worlds (Ueland, Dysvik et al., 2020). Receiving a cancer diagnosis and undergoing cancer treatment can be such a “break,” with the result that one no longer feels at home in life (Ueland, Dysvik et al., 2020).

Suffering also manifests obviously in an ongoing inner struggle, trying to come to terms with its limitations and with oneself. Hvidt (2017) found that cancer survivors live in an existentially disruptive movement in their lives according to their potential for becoming, transformation, and homecoming. In our study we see those participants after many years still struggling with transformation and homecoming. It seems like several processes of transition are restricted in some way. According to Meleis et al. (Meleis et al., 2000), transformation is the passage from the experience of suffering to the experience of health and wholeness. In this context, transition refers to a movement from one state, condition or place to another (Meleis et al., 2000). Holding oneself together as a whole entity (Eriksson, 2018) through the passage towards wholeness is challenging (Meleis et al., 2000). In our study transition is ongoing and challenging as life power and vitality is curtailed by cancer treatment.

Due to the loss of life power, there seems to be an ongoing inner struggle trying to find a way to handle and endure everyday life within this limitation. Morse (2001, 2011) describes two broad behavioural states identified in suffering, endurance and emotional suppression. Moreover, enduring suffering is the starting point towards transition (Morse, 2011). In our study, a response to the integrity of self, trying to come to grips with a situation but without really experiencing relief from suffering, seems to be the first stage of enduring. This process is consistent with Morse’s (2001) descriptions.

According to Knox (2020a), cancer survivors’ suffering is woven into a deep sense of rupture of self, and the struggle seems to be facing the unique task of self-becoming. According to Wood (2018, p. 151), ”[t]ransition survivorship is a passage through time and space” towards a new normal, re-entering a self, forever changed in a lifelong process (Wood, 2018). As such, the participants’ inner struggle to endure everyday life is to adapt to a narrower and more limited sphere of life. It seems to us that they try to accept the limitations and find their subjective meaning in the new delimited narrow life space.

In addition, suffering encompasses bearing the burden alone as the participants in our study lack confirmation of their personal changes. The burden they express seems to be losing a foothold in life and searching for one’s new self. This inner experience is difficult to share with others. In this way, our study illuminates the experience of the difficulties in expressing their suffering. This is in line with Adelbratt and Strang (2000) who claim that suffering persons are often missing the vocabulary necessary to express their deepest feelings. This inward and silent suffering indicates that participants try to handle their life situation as best they can by themselves. The participants seldom tell others how they feel and when no one is listening, it is a lonely suffering, kept inside oneself. It seems also to be somewhat inappropriate, that they are sad and feeling down when they have survived a cancerous disease. Furthermore, their suffering challenges societal norms, which increases the severity of the suffering experience. Additionally, suffering patients sometimes feel they need to protect others from their suffering and therefore suppress it consciously, which has been called “doubled suffering” (Langegard & Ahlberg, 2009).

Further theoretical interpretation and discussion point towards existential experiences as a movement between suffering and enduring as well as movement between alienworld and homeworld. These experiences of transition encompass an intermediate state. This is understood to be a multifaceted suffering expressed as holding back engagement, feeling exhausted, living more restricted, carrying the burden alone, and experiencing lack of resonance in one’s surroundings. Clearly there seems to be a struggle to endure, finding a foothold and lessening the burdensome situation.

Meleis et al. (2000) point towards possibilities of a transition process, which means a movement from one condition to another. Morse (2001) also highlights a way forward towards emotional release, enduring suffering in different stages. Leaving an intermediate state implies incorporation of new knowledge, altered behaviour, and a modified view of oneself and one’s situation (Meleis et al., 2000).

Steinboch describes such processes of becoming as a challenging shift, a critical vulnerable space where the unknown meets the familiar, and a safe space, striving to bring the two lifeworlds together into an alienworld–homeworld (Steinbock, 1994, 1995). The participants may be struggling with acknowledging the foreign world left by their cancer treatment and dwelling on their homeworld experience of life before their cancer diagnosis. In our study, participants are on a vague journey towards recognizing their alienworld, which is foreign and different from their homeworld, further striving to interweave these two worlds in present life. In achieving both alienworld and homeworld by understanding more of the intermediate state they live in; they might renew their homeworld experience but incorporate the alienworld to a greater extent. This involves a “liminal” experience of having to navigate the borderless and unfamiliar terrain between alienworld and homeworld until one reaches a transformation in which meaning ultimately stems from both lifeworlds (Hvidt, 2017; see also Ueland, Dysvik et al., 2020). The intermediate state incorporates these two lifeworlds, suffering–enduring, alienworld–homeworld, and needs both to be acknowledged.

As we see it, the participants are more focused on a balanced life rather than on a renewing life. This may be related to the fact that transition processes depend on interactions with the outside world, with relationships, and cannot only take place on an inner level. Meleis et al. (2000, p. 347) describes “living in limbo” as a phase of a health–illness transition characterized by loneliness. In this position there is clearly a need for a sensitive dialogue with others to endure and release suffering.

The study “‘Cured’ but not ‘Healed’, ”argues for a need in a “cure dominant” Western society health-care system, for a framework of care facilitating the transition from suffering to the experiences of wholeness (MacDonald et al., 2021). Studies suggest that the medical, disease-oriented approach, which is obviously necessary in cancer treatment to save lives, may overshadow the fact that “the whole person” needs help. The term “cancer survivor” might be problematic and can cause failure to recognize the vulnerability and challenges the person experiences in everyday life. Cultural expectations of gratitude for being alive might hinder a person’s honest and actual expressions (Ellingson & Borofka, 2020; see also Ellingson, 2017). Ellingson (2017) points towards telling an alternative story of cancer survivorship as “a new normal,” in a dialectic between celebrating the successful conclusion of cancer treatment with facing the tough realities of ongoing late effects.

### Methodological considerations

The strength of our study is that the interviews include both genders. This was men and women of different ages who had experienced different cancers and treatments, expressing one’s subjective suffering after finished cancer treatment. The information power appears to be strong due to sample specificity of 21 participants, quality of dialogue descriptions based on the study’s narrow aim, and using established theories (Malterud et al., 2016). Several theoretical perspectives serve to extend the existing knowledge beyond the empirical interview data theories (Malterud et al., 2016). Our study provides an important contribution to the understanding of life after cancer treatment, by focusing a lifeworld perspective on cancer survivors’ suffering. As such, the validity seems to be strong as the participants’ openness to describing in detail their lived experiences of suffering. A limitation of our study might be that we do not relate the suffering experience in detail stratified according to cancer type, treatment, late effects, side effects, or enduring damage. Rather, our study’s aim was to go beyond the biomedical perspective to achieve a lifeworld approach. We suggest that our findings are applicable to cancer survivors, and perhaps the study also contributes universal knowledge to other suffering contexts, which lead to major changes in life without the opportunity to return to where one was.

### Implications for practice

- There is a need to acknowledge the multifaceted suffering experienced in rehabilitation offered.
- It is significant that health professionals who meet survivors in different professional arenas might assist the person’s effort to handle suffering by searching for a way to live with suffering–enduring, alienworld–homeworld.
- Health professionals need to have insight into the intermediate state of living, allowing articulation and expression of thoughts and feelings, if necessary, for a long time.
- Opening up new knowledge of the lived experiences of suffering for cancer survivors in society might ameliorate the release transformation of suffering towards greater wholeness for survivors.

## Conclusion

A multifaceted and complex suffering left behind by completed cancer treatment is revealed as being like in a process of transition, in an intermediate state, as movement between suffering and enduring and between alienworld–homeworld. Living in an intermediate state after completed cancer treatment seems more to balance rather than renew one’s life space. Persons completing cancer treatment have existential suffering that is inadequately met by the current health-care system. Cancer survivors need to obtain a deeper understanding of their own suffering as being caught in an intermediate state. Our study provides an existential holistic perspective on suffering that may fill a gap in the current health-care rehabilitation to humans living beyond cancer. It is our opinion that the dominant biomedical discourse fails to capture the whole suffering that cancer survivors experience in their embodied lived lifeworld. New approaches are needed to confirm and support transformational processes by giving room for telling one’s own story. We believe that the health-care system needs to turn towards a lifeworld perspective throughout a patient’s cancer journey, listening to how the illness experiences have been integrated into their lifeworld and how suffering is experienced in everyday life.

A new and broader professional perspective is needed for the establishment of rehabilitation services in which health-care professionals care for the multifaceted experiences of suffering. This means a shift in focus from biomedical symptoms towards understanding of existential meaning for the person.
